# Survival analysis of the duration of rumors during the COVID-19 pandemic

**DOI:** 10.1186/s12889-024-17991-3

**Published:** 2024-02-19

**Authors:** Xiaoyan Liu, Lele Zhang, Lixiang Sun, Ran Liu

**Affiliations:** 1 School of Languages and Communication Studies, Beijing Jiaotong University, Beijing, 100044 China; 2 School of Medical Humanities and Management, Wenzhou Medical University, Wenzhou, 325035 China

**Keywords:** Rumor, Duration, COVID-19, Survival analysis

## Abstract

**Background:**

The emergence of the COVID-19 pandemic towards the end of 2019 triggered a relentless spread of online misinformation, which significantly impacted societal stability, public perception, and the effectiveness of measures to prevent and control the epidemic. Understanding the complex dynamics and characteristics that determine the duration of rumors is crucial for their effective management. In response to this urgent requirement, our study takes survival analysis method to analyze COVID-19 rumors comprehensively and rigorously. Our primary aim is to clarify the distribution patterns and key determinants of their persistence. Through this exploration, we aim to contribute to the development of robust rumor management strategies, thereby reducing the adverse effects of misinformation during the ongoing pandemic.

**Methods:**

The dataset utilized in this research was sourced from Tencent's “Jiao Zhen” Verification Platform's “Real-Time Debunking of Novel Coronavirus Pneumonia” system. We gathered a total of 754 instances of rumors from January 18, 2020, to January 17, 2023. The duration of each rumor was ascertained using the Baidu search engine. To analyze these rumors, survival analysis techniques were applied. The study focused on examining various factors that might influence the rumors' longevity, including the theme of the content, emotional appeal, the credibility of the source, and the mode of presentation.

**Results:**

Our study's results indicate that a rumor's lifecycle post-emergence typically progresses through three distinct phases: an initial rapid decline phase (0–25 days), followed by a stable phase (25–1000 days), and ultimately, an extinction phase (beyond 1000 days). It is observed that half of the rumors fade within the first 25 days, with an average duration of approximately 260.15 days. When compared to the baseline category of prevention and treatment rumors, the risk of dissipation is markedly higher in other categories: policy measures rumors are 3.58 times more likely to perish, virus information rumors have a 0.52 times higher risk, epidemic situation rumors are 4.86 times more likely to die out, and social current affairs rumors face a 2.02 times increased risk. Additionally, in comparison to wish rumors, bogie rumors and aggression rumors have 0.26 and 0.27 times higher risks of dying, respectively. In terms of presentation, graphical and video rumors share similar dissolution risks, whereas textual rumors tend to have a longer survival time. Interestingly, the credibility of the rumor's source does not significantly impact its longevity.

**Conclusion:**

The survival time of rumors is strongly linked to their content theme and emotional appeal, whereas the credibility of the source and the format of presentation have a more auxiliary influence. This study recommends that government agencies should adopt specific strategies to counter rumors. Experts and scholars are encouraged to take an active role in spreading health knowledge. It's important for the public to proactively seek trustworthy sources for accurate information. Media platforms are advised to maintain journalistic integrity, verify the accuracy of information, and guide the public towards improved media literacy. These actions, collectively, can foster a collaborative alliance between the government and the media, effectively combating misinformation.

## Introduction

At the end of 2019, the sudden outbreak of the COVID-19 pandemic created a significant upheaval, presenting an unparalleled challenge to global health and safety. By February 2, 2020, the World Health Organization had recognized the concurrent rise of an “infodemic”, accompanying the swift spread of COVID-19. This infodemic was characterized by an excessive amount of information, complicating the task of differentiating factual information from falsehoods. Social media platforms amplified the panic, resulting in the rapid dissemination of false information at an unprecedented rate. In many countries, the general public is frequently confronted with an abundance of misinformation, compounded by restricted access to trustworthy information sources and a deficiency in suitable guidance [1–3]. As a result, the public faced significant challenges in accessing credible information sources and obtaining accurate guidance during this turbulent period. This era was marked by the rampant spread of online rumors, including unfounded claims like “smelling onions can prevent COVID-19” and “all supermarkets and pharmacies will close in three days”. These baseless rumors flooded social media platforms, intensifying public panic, destabilizing society, and severely hindering the population's capacity to make informed decisions regarding the pandemic. Such misinformation significantly hampered the implementation of effective strategies to combat the spread of COVID-19.

The investigation into rumors has its roots deeply embedded in psychology. Knapp's theory posits that rumors are crafted for believability, are pertinent to ongoing events, and are widely circulated without validation from official sources [4]. Building on Knapp's insights, Allport and Postman laid the groundwork in the field of rumor psychology, defining rumors as assertions or beliefs spread via interpersonal communication without corroborative evidence [5]. They formulated the seminal equation for rumor propagation: Rumor = Importance × Ambiguity, suggesting that the breadth of a rumor's spread is proportional to its relevance and the uncertainty surrounding the associated facts. Subsequent research by scholars from diverse disciplines extended the study of rumors beyond psychology, adopting a more encompassing social perspective. The Project Revere experiment revealed that during the course of interpersonal diffusion, messages experience leveling, sharpening, and assimilation, leading to their distortion and inaccuracy [6]. This experiment not only reinforced Allport and Postman's transmission equation but also explored the influence of interpersonal networks and media in the spread of rumors. With technological progression, the internet and network-based rumors have opened new avenues and presented challenges for researchers. Online rumors proliferate more rapidly, reach a broader audience, and have a greater impact, while the gathering of rumor-related data has become more feasible. Researchers have begun creating models to simulate rumor spread [7], with goals to detect and identify rumors [8], trace their origins [9], and predict their peak popularity and dissipation times [10]. Some have compared rumor spread to viral transmission, equating it to a social “intellectual epidemic” [11].

The COVID-19 pandemic has presented a prolific landscape for exploring the dynamics of rumors, given the significant proliferation of such unverified information throughout this global crisis. Regarding the rumors that emerged during the COVID-19 epidemic, numerous scholars have conducted extensive research from various perspectives. Several studies have focused on categorizing rumors specific to the COVID-19 pandemic [12], offering customized strategies for each identified category [13]. Other researchers have continued in the established tradition of creating predictive models to analyze the transmission patterns of rumors and to explore the effects of various factors on the spread of these rumors [14].

For instance, in their comparative analysis of COVID-19 related rumors from two prominent rumor debunking platforms, China's “Jiao Zhen” and the UK's Full Fact, Liu and Zuo [15] employed a novel topic mining model to uncover the thematic distribution and evolution of online rumors. Focusing specifically on the antecedents related to rumor characteristics, Ning et al. [16] conducted a descriptive research study to analyze the traits and dissemination of rumors during the early stages of the COVID-19 pandemic in China. Their investigation included an assessment of the effectiveness of countermeasures, such as corrective announcements by health authorities. They discovered a positive correlation between the volume of rumor circulation and the severity of the pandemic, noting that the release of corrective information significantly reduced the proportion of rumors compared to accurate reporting.

In a comprehensive study spanning 2006 to 2017, Vosoughi et al. [17] scrutinized the dissemination of all verified true and false news stories on Twitter. Their findings indicated that false information spread more broadly, rapidly, deeply, and widely than truthful information, with the impact of false political news being especially significant in comparison to other categories like terrorism, natural disasters, science, urban myths, or financial information. They also observed that fake news tended to be more novel than real news, suggesting a higher likelihood of novel information being shared. Song et al. [18] explored the influence of rumor types, content attributes, and source characteristics on the spread of COVID-19 rumors, drawing data from Sina Weibo, a major Chinese social media platform. Similarly, Chen et al. [19] compared the rumors circulating in different phases of the COVID-19 pandemic using Chinese microblogging platforms. Their study delved into the distinct impacts on rumor propagation, considering factors such as the mode of media presentation, the original platform of the rumor, and the type of entity refuting the rumor.

In addition to intrinsic aspects of rumors, such as thematic content, presentation style, and source origin, various scholars have also investigated the precursors of rumor spread through the lens of public characteristics. For instance, Luo et al. [20] examined the factors influencing rumor propagation, focusing on peer dynamics and self-perceived health efficacy. Their research, grounded in online survey data, uncovered that peer interactions and the perceived condition of peers heightened COVID-19 related fears, subsequently fueling the dissemination of online rumors. Conversely, a strong sense of health self-efficacy was found to mitigate the influence of COVID-19 fears on the spread of these rumors. Extending this line of inquiry, Ding and Luo [21] applied the behavioral immune system theory to assess the impact of perceived infectivity on rumor transmission during the COVID-19 crisis, exploring the mediating role of trust in rumors.

Current research on rumors predominantly concentrates on delineating their transmission dynamics or investigating the influence of factors related to the rumor itself or the public on their spread, often employing traditional research methodologies. However, the aspect of the lifespan of rumors has rarely been the central focus of study. While some rumors adhere to a “Seven-day Law of Propagation”, mirroring the fleeting nature of online trending topics by appearing and vanishing swiftly, there exist persistent rumors that subtly influence people's perceptions, emotions, and health. An in-depth analysis of the survival time of rumors can provide valuable insights into the distribution and propagation patterns of various types and categories of rumors. Such understanding is crucial for enhancing governmental capabilities in predicting, identifying, and managing rumors, ultimately mitigating their adverse impacts on society.

Therefore, this article proposes two research questions:

- Research Question 1: How is the distribution of COVID-19 rumor survival time in online networks during the pandemic?
- Research Question 2: Which factors exert influence on the survival time of COVID-19 rumors in online networks during the pandemic, and in what ways do these factors contribute to the longevity of these rumors?

## Materials and methods

### Data source

In this research, Tencent News' “Jiao Zhen” platform was instrumental as a primary data source. Initiated in January 2017, “Jiao Zhen” represents the first fact-checking initiative in China, aligning with the rigor and precision that characterize public health research [22]. It is distinguished as one of a mere quintet of active Chinese sites in the Duke Reporters Lab's global fact-checking web database [23]. This platform leverages big data analytics, employing a refined weighting methodology to assess rumors based on their relevance, obscurity, propagation potential, and potential harmful impact. Incorporating state-of-the-art technologies such as deep learning, image recognition, and algorithms for popularity assessment, “Jiao Zhen” is dedicated to the accurate identification and debunking of rumors [24]. Mirroring the goals of its American counterparts like PolitiFact and FactCheck.org, “Jiao Zhen” aims to effectively counter the spread of misinformation and enhance public discernment between factual and misleading information, a crucial aspect in public health information dissemination.

The dataset used in this study comprises pandemic-related rumors and was extracted from Tencent's “Jiao Zhen” Fact-Checking Platform, specifically the "Real-Time Debunking of COVID-19 Rumors" system. This encompasses a diverse array of pandemic-related rumors, examples of which include claims of “Zhengzhou residents being forcibly quarantined for non-compliance with lockdown measures”, and “An aunt in Shanghai brought blankets from the makeshift hospital back to the community, causing multiple people to become infected”. The observational duration of our study was three years, extending from January 18, 2020, to January 17, 2023. Following a comprehensive process of cleaning and deduplication, we successfully collated a dataset comprising 754 instances of rumor data. During the data processing phase, the primary focus was on eliminating duplicate rumors and those unrelated to the COVID-19 pandemic.

### Dependent variable

For effective survival analysis, defining the event's duration, or “survival time”, is crucial. The start time marks the period before the target event's occurrence, and the end time signifies the point when the risk set ends. The interval between these two points is the event's survival time [25]. Accurate assessment hinges on detailed information about specific rumors online, with relevant internet posts being key indicators [26].

Extracting temporal data from textual online sources is achievable through the use of web crawling techniques on search engines. This approach is fundamental in collecting extensive data necessary for conducting thorough survival analysis. By employing web crawlers, researchers can systematically gather and analyze the publication and expiration dates of online content, thereby determining the survival time of events or phenomena reflected in these digital texts.

This consensus involves identifying the timepoints of both the first and last occurrences of a rumor as the respective start and end markers. For instance, Kwon, et al. [27] in their exploration of rumor spreading patterns across different timeframes, identified the date of the initial appearance of rumors on Twitter as a crucial reference point. Additionally, Lee et al. [28] studied the diffusion of rumors and non-rumors on Twitter during extreme events, considering the time difference between the first tweet and the event time as the response time. Similarly, Bodaghi and Goliaei [29] determined the duration of a rumor dataset in their collection by identifying the first and last tweets associated with the topic. Burnap et al. [30] utilized survival analysis, explicitly stating that the determination of a rumor's survival time in their study was based on the duration between the first and last retweets. Our methodology closely aligns with these approaches, which guided us to adopt a similar method in our research.

For determining the numerical value of the dependent variable, the rumor survival time, we used the Baidu platform for data collection. Baidu is one of the largest search engines in the world [31], particularly in China, Baidu is the most-used search engine [32]. As of December 2023, it accounted for 66.52% of Search Engine Market Share in China [33]. Rumors related to the pandemic in China are also searched in Chinese; therefore, this paper has chosen to collect the dependent variable data on rumors through Baidu searches. In this study, the titles of various rumors from the Tencent News' “Jiao Zhen” platform were used as search keywords for individual searches in Baidu.

Specifically, we crawl all search results from the Baidu search engine and arrange them in ascending order based on their timestamps. The earliest appearance of the rumor is identified as the start time (N_0_), while the latest appearance is recognized as the death time (N_1_). The duration of each rumor was determined based on the time interval between these two points. The survival time of the rumor is determined by subtracting the start time (N_0_) from the death time (N_1_). To ensure the accuracy of the time information, we meticulously filter out content that is erroneously copied or sourced from delayed plagiarized material on zombie websites.

### Independent variables

The research into the factors affecting rumor dissemination has extensively concentrated on multiple dimensions, including the content theme, the emotional demands of users, the reliability of information sources, and the forms of presentation. Through a comprehensive review of existing literature, this study has pinpointed independent variables that play a crucial role in the spread of rumors. These identified variables provide a deeper insight into the mechanics of rumor propagation, offering a solid foundation for developing more effective strategies to manage and counteract the spread of misinformation.

#### Content theme

In the vast realm of internet information, certain topics attract substantial attention, becoming prominent on the social agenda for prolonged periods [34]. When examining COVID-19 rumors, scholars have categorized them based on their content.

Alongside this, the spread of rumors, especially in the context of health-related misinformation, has been a long-standing subject of academic inquiry. Key areas impacted by rumors include public health, disease, and dietary health. Li et al. [35] emphasized the importance of recognizing hot topics in health rumors, particularly those disseminated through platforms like the WeChat Official Account. The categorization of rumor types plays a crucial role in understanding their dissemination. Tai and Sun [36] classified rumors related to SARS into four thematic categories: legendary rumors, aetiological narratives, proto-memorates, and bogies. In the context of COVID-19, the typology of rumors has been more extensively developed. Islam et al. [37] divided COVID-19 vaccine rumors into ten categories: COVID-19 vaccine development, availability, and access; vaccine-related morbidity and mortality; political and economic motives; safety, efficacy, and acceptance; COVID-19 susceptibility due to exposure to other vaccines; vaccine reagents; mandatory vaccines and ethics; vaccine alternatives and necessity; conspiracy theories; and miscellaneous.

Similarly, Yang et al. [38] categorized COVID-19 rumors into six categories, encompassing viral research, pandemic prevention and control, confirmed cases, overseas epidemic situations, social welfare, and government actions.

Building on the categorization framework proposed by Chen et al. [39], this study refines and adapts their classification to better address the unique characteristics of COVID-19 rumor propagation. The revised scheme encompasses five categories: prevention and treatment, policy measures, virus information, epidemic situation, and social current affairs.

Prevention and Treatment: “Curative medicine and preventive methods of COVID-19” [39]. It covers incorrect or misleading information about medications, health practices, and remedies that are claimed to be effective in preventing or curing COVID-19. For instance, Hydroxychloroquine combined with Azithromycin can treat COVID-19.

Policy Measures: It includes “returning to normal production and life,” “international and domestic travel restrictions” and “policies for dealing with COVID-19”. “The date and schedule of restoring schooling, production, and living”, “policies of travel restriction issued by China’s government and other countries’ governments” and “official policies responding to COVID-19 issued by China’s multi-level governments” [39]. It includes false or distorted information about lockdowns, social distancing rules, mask mandates, vaccination policies, and other public health interventions. For instance, there is an online rumor that Dandong will be locked down on June 26^th^.

Virus Information: It includes both “conspiracy theories” and “epidemiological characteristics of COVID-19”. “Related epidemiological characteristics” and “conspiracy theory associated with the virus source or biological warfare” [39]. It encompasses incorrect or misleading facts about the nature and behavior of the virus. For instance, there is a claim that flying catkins can accelerate the spread of the COVID-19 virus.

Epidemic Situation: “Severe situations and reappearances of COVID-19” [39]. This includes inaccurate or exaggerated information about infection rates, death rates, the number of cases, hotspots, and the overall severity and spread of the pandemic. For instance, there is a claim that over 300 cases of COVID-19 infection have emerged in Nanjing.

Social Current Affairs: “Fake news related to social life” [39]. It includes false narratives or misleading information about the pandemic's effects on various aspects of social life, such as economic downturns, social unrest, cultural impacts, and other societal consequences of the pandemic. For instance, there is a claim that the Italian government is disposing of COVID-19 victims' bodies into mass graves.

#### Emotional needs

Rumors, according to Knapp's book “A Psychology of Rumor”, are driven by the fulfillment of certain emotional needs. These needs can be categorized into wish rumors, bogie rumors, and aggression rumors [4]. Building on this framework, Yang and Paek found that during risk outbreaks, bogie rumors and aggression rumors were the most prevalent [40]. Similarly, Paek and Hove analyzed rumors related to food contamination by nuclear radiation in Fukushima, Japan, and discovered that aggression rumors had a stronger impact in terms of belief and transmission intentions than bogie rumors [41]. The categorization of the “emotional needs” variable in this study is informed by the research of Yang and Paek [40], Paek and Hove [41], and Knapp [4]. In line with Knapp's categorization of rumors based on emotional needs, this article classifies the collected rumors into wish rumors, bogie rumors, and aggression rumors.

Wish Rumors refer to rumors that express “the wishes and hopes of those among whom they circulate” [4]. For instance, the rumor “soybeans boiled in water can reduce fever” subtly expresses the collective desire for an easy cure to COVID-19, and thus can be categorized under 'Wish Rumors'. These rumors often convey positive emotions, expressing people's longing for ideal future scenarios.

Bogie rumors refers to “the precise opposite of the pipe-dream rumor” [4]. For example, the rumor “Omicron is like minor AIDS, and repeated infections lead to immune system failure” manifests the public's fear of the unknown aspects of the disease, reflecting widespread panic about the spread of the pandemic. It reflects people's apprehension about danger, often garnering societal attention and triggering a widespread panic response within the community.

Aggression rumors are characterized as rumors that possess destructive and aggressive qualities [4]. For example, the rumor “An elderly woman in Tianjin is using face masks to lure and abduct children” stigmatizes the elderly, eroding trust among people and creating panic within the community. These rumors are inherently designed to fragment groups and foment discord, thereby exerting a deleterious effect on the harmony and stability within social collectives. The unchecked proliferation of such rumors poses a significant threat to the orderly and tranquil progression of society, undermining its foundational cohesive elements.

#### Source credibility

The role of source credibility in rumor dissemination is significant, as demonstrated by Hovland et al. [42], who discovered that more credible sources wield greater persuasive power, whereas less credible ones have a reduced effect. Bordia and Difonzo's experiment on the “computer virus” rumor further supports this, showing that when the refutation source was changed from a “fellow student” to the “Computer Incident Advisory Capability”, the perceived professionalism and trustworthiness of the source increased [43]. In the digital realm, users often assess information credibility based on their trust in the publisher [44]. Influential and authoritative online figures significantly affect the velocity and extent of rumor propagation [45], with more credible publishers facilitating faster spread [46]. Liu et al. 's research indicates that source credibility, along with expertise and attractiveness, greatly influences the likelihood of information being shared [47].

Despite often lacking verifiable sources, rumors frequently incorporate references to seemingly authoritative entities to boost their believability. Rumor spreaders may deliberately use fabricated but reliable-looking sources to lend credibility to their information [48]. Therefore, this study categorizes sources as high credibility when they are authoritative institutions known for accurate reporting [49], or when they are trustworthy, reputable, or popular celebrities [50]. Conversely, rumors not referencing authoritative institutions or celebrities are deemed to originate from low credibility sources.

#### Presentation format

The format and dimensions of information presentation are key in assessing information quality [51]. Different formats such as text, images, and videos each uniquely influence the readability and intuitiveness of content. Images and videos, with their visual appeal and straightforward nature, meet internet users' desire for vivid, on-site event representation. Links offer an additional dimension, directing readers to external web pages for further information, thus enriching the content's format [7].

In light of these considerations, this study categorizes the primary formats of rumor presentation into three distinct groups: text, image, and video. Rumors presented in the form of chat logs or written articles are classified as “text”. Those primarily using images for support are categorized as “image". Rumors disseminated through video content are labeled as “video”. The coding details can be found in Table 1.

**Table 1:** Variable coding

Variable	Meanings	Coding
X_1_	Content theme	1. Prevention and treatment
		2. Policy measures
		3. Virus information
		4. Epidemic situation
		5. Social current affairs
X_2_	Emotional need	1. Wish rumor
		2. Bogie rumor
		3. Aggression rumor
X_3_	Source credibility	0. Low credibility source
		1. High credibility source
X_4_	Presentation format	1. Text
		2. Image
		3. Video
Time	Duration	Continuous variable
Event	Status	0. Censoring
		1. Death

During the coding phase of this study, two coders were employed, and to ensure reliability, an intercoder reliability test was conducted. This test involved randomly selecting 10% of the rumor data for analysis. The resulting Kappa coefficients, which ranged from 0.874 to 0.938, demonstrated a high level of consistency between the coders. These values indicate that there was a strong agreement in their coding decisions, achieving a level of agreement that is considered satisfactory for research purposes. This rigorous approach to coding enhances the reliability and validity of the study's findings.

### Survival analysis

This paper employs survival analysis methods, a statistical approach used to analyze the occurrence and timing of events within a specified period. This method does permit certain cases to be omitted [52].

Survival analysis methods can elucidate why certain individuals are at a heightened risk of encountering specific events of interest [53]. This approach effectively addresses two challenges not typically resolved by traditional multivariate statistical methods: censoring and time-dependent covariates [53]. A hazard model, a key component of survival analysis, adeptly manages censored observations, which contain only partial information, and accommodates covariates that fluctuate over the observation period. These unique characteristics distinctly set it apart from other regression models [53, 54].

Originally developed for use in biology and medicine, survival analysis has progressively broadened its scope of application. It has been effectively applied in various fields, including estimating customer lifetime value [55], modeling technology adoption [56], analyzing information streams [30], and investigating trending topics on social media platforms [57]. These applications are predominantly found in management science, engineering, and information science. However, its use within journalism and communication studies remains comparatively underexplored. To address this gap, our study incorporates the Kaplan‒Meier (KM) survival estimate and the Cox proportional hazards model, applying these within the broader framework of survival analysis.

## Results

### Univariate analysis of duration

In this research, the Kaplan–Meier survival estimate, a robust univariate method for survival analysis, was utilized to examine the relationship between various independent variables and the survival time of rumors. By conducting separate analyses with different independent variables on the rumor data, we derived the distribution of survival times and statistical outcomes, as detailed in Table 2.

**Table 2:** Kaplan‒Meier test of factors

Factors	Log Rank (Mantel‒Cox)			Breslow (Generalized Wilcoxon)		
	**Chi-Square**	**df**	**Sig**	**Chi-Square**	**df**	**Sig**
Content theme	319.956	4	.000	264.357	4	.000
Emotional need	85.140	2	.000	51.495	2	.000
Source credibility	.536	1	.464	.028	1	.866
Presentation format	14.104	2	.001	6.462	2	.040

Statistically, the log rank test is designed to highlight long-term effects and treats each time point equally. On the other hand, the Breslow test is more focused on short-term effects and assigns weights based on the number of cases at each time point. The analysis of Table 2 reveals significant disparities (*p* = 0.000 < 0.05) in the survival times of rumors across five content themes, as indicated by both the log rank and Breslow tests. Likewise, the survival curves for rumors categorized by emotional needs and presentation format also show significant differences, with significance levels falling below 0.05.

However, the influence of source credibility on rumor survival time presents a different picture. The results from both the log rank test (*p* = 0.464 > 0.05) and the Breslow test (*p* = 0.866 > 0.05) were not statistically significant, suggesting that the credibility of the source does not have a substantial impact on the duration for which a rumor persists. This finding underscores the complexity of factors influencing rumor longevity and highlights the need for further exploration in this area.

Table 3 offers an extensive breakdown of the number of rumors, complete data counts, censored data counts, and the ratio of censored data to complete data across different classification dimensions.

**Table 3:** Summary of rumor cases

	**Total N**	**Percent**	**N of Events**	**Censored**	
				**N**	**Percent**
**Content theme**					
Prevention and treatment	218	28.91%	191	27	12.39%
Policy measures	266	35.28%	264	2	0.75%
Virus information	60	7.96%	53	7	11.67%
Epidemic situation	85	11.27%	84	1	1.18%
Social current affairs	125	16.58%	121	4	3.20%
**Emotional need**					
Wish rumor	266	35.28%	245	21	7.9%
Bogie rumor	233	30.90%	218	15	6.4%
Aggression rumor	255	33.82%	250	5	2.0%
**Source credibility**					
Low credibility source	524	69.50%	493	31	5.9%
High credibility source	230	30.50%	220	10	4.3%
**Presentation format**					
Text	501	66.45%	467	34	6.8%
Image	115	15.25%	111	4	3.5%
Video	138	18.30%	135	3	2.2%
**Overall**	754	100%	713	41	5.44%

In the category of content theme, policy measures rumors were most prevalent, with a total of 266 cases, making up 35.28% of all rumors. Prevention and treatment rumors followed, comprising 218 cases or 28.91% of the total, underscoring the widespread nature of rumors related to policy actions and health measures. In contrast, virus information rumors were less frequent, amounting to only 60 cases or 7.96% of the total. Rumors about the epidemic situation and social current affairs accounted for 85 (11.27%) and 125 (16.58%) cases respectively, highlighting the variety in rumor topics.

Concerning emotional demands, wish rumors topped the list with 266 cases, constituting 35.28% of all rumors. Bogie rumors, with 255 cases, represented 33.82%, while aggression rumors numbered 233, accounting for 30.90% of the total. These numbers suggest a fairly even distribution of rumors across different emotional contexts.

In terms of source credibility, rumors from low credibility sources were predominant, totaling 524 cases or 69.50% of all rumors. This is more than double the number from high credibility sources, which accounted for 230 cases or 30.50%. This stark difference indicates that most rumors are not associated with authoritative entities or celebrities, highlighting the prevalence of unverified information.

Looking at presentation format, text-based rumors were the most common, with 501 cases or 66.45% of the total. Image-based and video-based rumors were less numerous, at 115 (15.25%) and 138 (18.30%) cases respectively. These findings reveal that despite the internet's capacity to distribute diverse types of content, text-based rumors remain the most widespread, often characterized by their ambiguous nature.

Table 4 presents an in-depth statistical analysis of the mean and median survival times for different rumor content themes, utilizing the Kaplan‒Meier method. The findings reveal that while half of the rumors dissipate within a relatively short timeframe of less than 25 days, the overall average survival time is significantly longer, at 260.15 days. This indicates the presence of some rumors that persist much longer, thus raising the average duration.

**Table 4:** Means and Medians for Survival Time

	**Mean**^**a**^				**Median**			
	**Estimate**	**Std. Error**	**95% Confidence Interval**		**Estimate**	**Std. Error**	**95% Confidence Interval**	
			**Lower Bound**	**Upper Bound**			**Lower Bound**	**Upper Bound**
**Content theme**								
Prevention and treatment	610.942	30.023	552.096	669.788	670.000	96.424	481.008	858.992
Policy measures	76.229	12.157	52.401	100.057	11.000	1.439	8.180	13.820
Virus information	362.402	55.435	253.749	471.055	80.000	73.336	.000	223.738
Epidemic situation	30.183	7.815	14.867	45.500	8.000	.921	6.194	9.806
Social current affairs	144.678	25.490	94.719	194.638	18.000	3.010	12.100	23.900
**Emotional need**								
Wish rumor	443.529	28.657	387.361	499.697	158.000	88.372	.000	331.210
Bogie rumor	191.640	22.408	147.721	235.559	18.000	2.747	12.615	23.385
Aggression rumor	126.885	16.234	95.066	158.703	19.000	1.802	15.468	22.532
**Source credibility**								
Low credibility source	262.585	17.577	228.135	297.036	25.000	3.465	18.208	31.792
High credibility source	253.975	25.166	204.648	303.301	24.000	3.475	17.189	30.811
**Presentation format**								
Text	300.188	18.891	263.163	337.214	29.000	5.037	19.127	38.873
Image	179.485	31.057	118.614	240.356	23.000	3.830	15.494	30.506
Video	181.802	26.691	129.488	234.116	21.000	3.915	13.326	28.674
**Overall**	260.153	14.427	231.875	288.430	25.000	2.460	20.178	29.822

^a^Estimation is limited to the largest survival time if it is censored

In terms of content themes, prevention and treatment rumors stand out with an average survival time of 610.94 days, markedly longer than that of epidemic situation rumors. The average survival time for virus information rumors is 362.4 days. Conversely, epidemic situation rumors have the shortest average survival time at 30.18 days, followed by policy measures rumors at 76.23 days. The median survival times, indicating when 50% of rumors have dissipated, also show notable differences. For example, half of the prevention and treatment rumors last over 670 days, while half of the epidemic situation rumors and policy measures rumors vanish within just 8 and 11 days, respectively.

Focusing on emotional needs, wish rumors have the longest average survival time at 443.53 days. Bogie rumors follow with an average of 191.64 days, and aggression rumors have an average of 126.89 days. The discrepancy is significant, with the survival time of wish rumors being about 3.5 times longer than that of bogie rumors. The median survival times reflect this trend, with half of the wish rumors persisting for over 158 days, compared to only 18 and 19 days for bogie and aggression rumors, respectively.

Regarding source credibility, the average survival times of rumors from low and high credibility sources are similar, around 260 days, and their median survival times are close to 25 days. This suggests that, regardless of source credibility, half of the rumors dissipate within a month, showing little difference between the two categories.

Lastly, in terms of presentation formats, text-based rumors have a significantly longer average survival time of 300.19 days, about 1.67 times that of image- and video-based rumors. However, the median survival times for all three formats are relatively close, between 20 and 30 days. This indicates that while a minority of text-based rumors last much longer, they considerably affect the overall average survival time for this category.

**Fig. 1:**
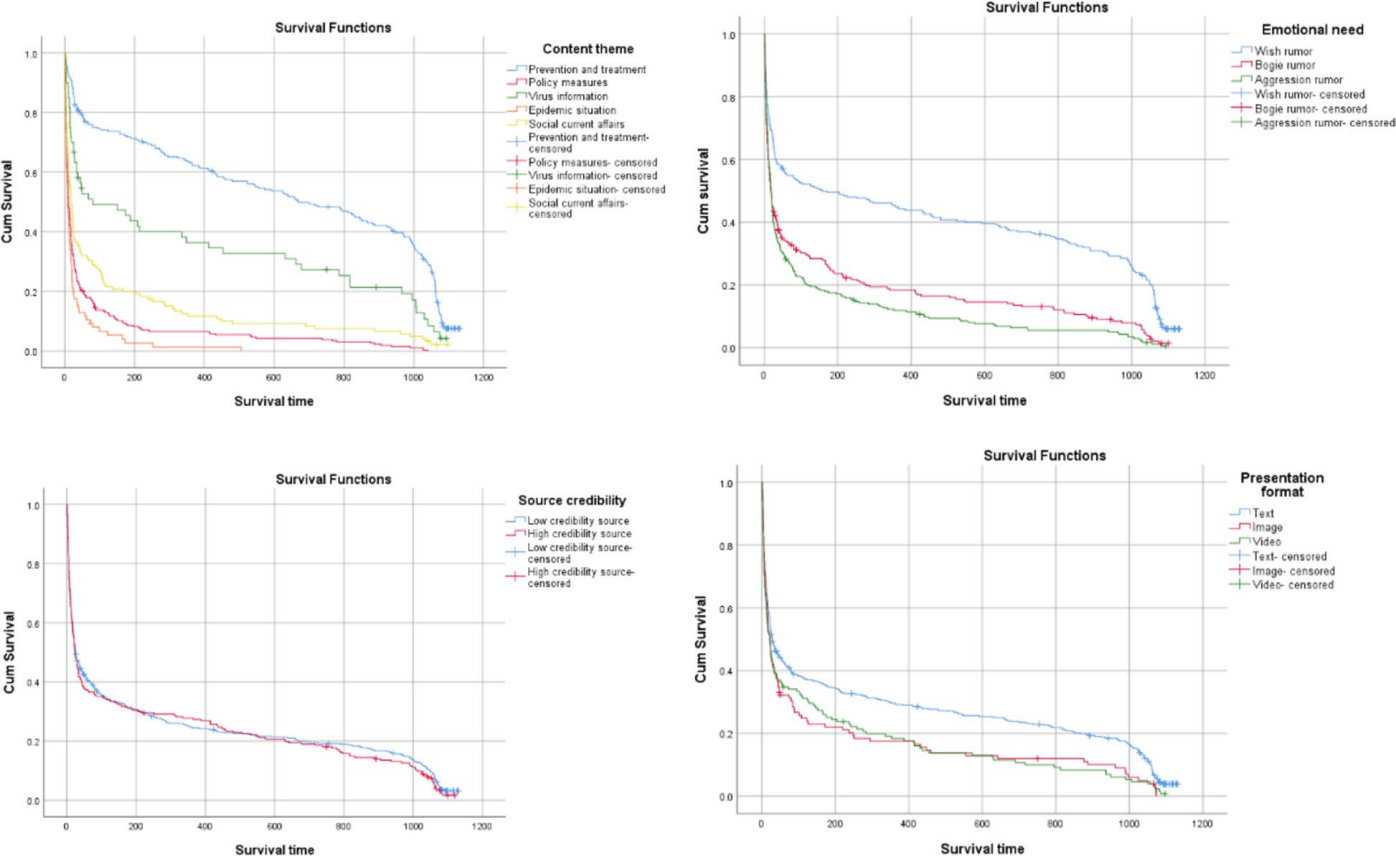
Survival function curve of rumors

Figure 1 presents the intriguing Kaplan–Meier (KM) survival curves for various categories of rumors, with the survival time plotted on the x-axis and the KM survival probability on the y-axis. Each curve is colorfully delineated to represent the survival functions of different variables. The discrete markers along these curves, reminiscent of fences, indicate the presence of censored data. From Fig. 1, it's observable that at the onset (near 0 survival time), the survival probability is close to 100%, gradually declining over time until it approaches 0.

In the early stages following a rumor outbreak, the KM survival curves exhibit a steep decline. This might be due to certain rumors having inherent weaknesses that allow for quick public debunking. Over time, the rate of decline in these curves becomes less pronounced. If there are noticeable differences in the distribution of these curves, it implies that the corresponding factor significantly influences the survival time.

Looking at the KM curves for different content themes, the curve for prevention and treatment rumors remains higher than others, indicating a slower rate of decline and a prolonged presence. Virus information rumors also display resilience, often cloaked in a scientific guise, making them harder for the general public to debunk due to required expertise. Rumors about epidemic situations and policy measures, often spurred by public speculation following a disease outbreak, exhibit similar survival times. The curve for epidemic situation rumors shows the sharpest initial decline, indicating these rumors are quickly clarified. Policy measures rumors also experience a rapid drop in survival probability.

Regarding emotional needs, the KM curves show that different emotional demands affect rumor longevity differently. Wish rumors have the highest survival probability, while bogie rumors are at the lower end. Aggression rumors, which often face quick rebuttals, have a tendency to fade swiftly. Both bogie and aggression rumors can trigger significant social reactions. If not addressed promptly, they can disrupt social order. Wish rumors, on the other hand, demonstrate staying power, offering psychological comfort during the epidemic.

The KM curves for rumors based on source credibility show multiple intersections, suggesting no significant difference in survival times between high and low credibility sources.

In terms of presentation format, significant differences are apparent. The curves for image and video rumors run parallel, but the curve for text rumors is consistently higher, indicating a longer survival. Image and video rumors, providing concrete evidence, can be more quickly debunked. Despite initial credibility, image and video rumors do not sustain long-term. This illustrates that while visually compelling, such rumors eventually succumb to fact verification and do not prolong the survival time of the rumor.

### Cox model results analysis

The Cox proportional hazards regression model, renowned for its analytical prowess, enables the concurrent evaluation of multiple variables' effects on survival time. In this study, variables such as content theme, emotional demand, source credibility, and presentation format were integrated as covariates for a holistic analysis.

Table 5 reveals the detailed results of the Cox model's coefficients, which are instrumental in assessing the model's fit. Initially, the -2 log-likelihood value for the null model, which excludes any independent variables, stands at 8196.354. However, the introduction of the independent variables leads to a significant change. Table 5 indicates the -2 log-likelihood value for the model with these variables is 7883.994, showing a substantial reduction of 312.36. Given the model's 9 degrees of freedom and a *p*-value close to 0.000, which is well below the standard significance threshold of 0.05, the results demonstrate marked statistical significance. This significant difference in the -2 log-likelihood values highlights the impact of the included variables on the survival time of rumors, affirming the model's effectiveness in analyzing these factors (See Table 5).

**Table 5:** Omnibus tests of model coefficients^a^

-2 Log Likelihood	Overall (score)			Change From Previous Step			Change From Previous Block		
	**Chi-square**	**df**	**Sig**	**Chi-square**	**df**	**Sig**	**Chi-square**	**df**	**Sig**
7883.994	318.321	9	.000	312.360	9	.000	312.360	9	.000

^a^Beginning Block Number 1. Method = Enter

The regression analysis in Table 6 offers critical insights into the Cox model's variables, including regression coefficients (B), standard error (SE), and Wald test results, which assess the significance of these coefficients. The Exp(B) value is especially pivotal as it represents the relative hazard ratio, indicating how a particular covariate alters the risk function compared to the baseline.

**Table 6:** Cox model results

	**B**	**SE**	**Wald**	**df**	**Sig**	**Exp(B)**	**95.0% CI for Exp(B)**	
							**Lower**	**Upper**
**Content theme**								
Prevention and treatment			209.035	4	.000			
Policy measures	1.522	.116	171.578	1	.000	4.580	3.648	5.752
Virus information	.418	.168	6.197	1	.013	1.519	1.093	2.111
Epidemic situation	1.769	.158	125.965	1	.000	5.863	4.305	7.985
Social current affairs	1.105	.135	66.895	1	.000	3.018	2.316	3.933
**Emotional need**								
Wish rumor			6.152	2	.046			
Bogie rumor	.231	.108	4.562	1	.033	1.260	1.019	1.558
Aggression rumor	.238	.105	5.113	1	.024	1.269	1.032	1.560
**Source credibility**								
High credibility source	.022	.084	.068	1	.794	1.022	.867	1.206
**Presentation format**								
Text			.884	2	.643			
Image	-.044	.112	.155	1	.693	.957	.769	1.191
Video	.073	.103	.502	1	.479	1.076	.879	1.317

When B is positive (greater than 0), Exp(B) exceeds 1, indicating an increase in the hazard function, thereby identifying the variable as a risk factor. Conversely, a negative B (less than 0) results in Exp(B) being less than 1, suggesting a decrease in the hazard function and thus classifying the variable as a protective factor. A B of 0 implies that Exp(B) equals 1, meaning the variable does not affect the hazard function.

From Table 6, it's evident that all content themes and emotional needs categories produced significant results (*p* < 0.05), indicating a substantial impact on survival time distribution within the model. Thus, both content theme and emotional need are influential in determining the survival time of rumors. However, the variables of source credibility and presentation format, with *p*-values above 0.05, are statistically insignificant as influencing factors.

The 95% confidence interval of Exp(B) for image and video formats within presentation format includes 1, showing no significant risk difference compared to text, the reference group. This finding diverges from the Kaplan–Meier method results and warrants further exploration. Similarly, the confidence interval for high credibility sources within source credibility also encompasses 1, indicating no significant risk difference between high and low credibility sources, consistent with the Kaplan–Meier findings.

In terms of content themes and emotional needs, positive regression coefficients suggest these are risk factors for rumor demise. For emotional need variables, policy measures rumors have a 3.58 times higher death risk than prevention and treatment rumors, virus information rumors a 0.52 times higher risk, epidemic situation rumors a 4.86 times higher risk, and social current affairs rumors a 2.02 times higher risk, all statistically significant. The hazard ranking for content themes is epidemic situation, policy measures, social current affairs, virus information, and prevention and treatment. When considering consistent content themes and using wish rumors as the reference, bogie rumors have a 0.26 times higher death risk, and aggression rumors a 0.27 times higher risk. The risk levels for emotional need categories are aggression, bogie, and wish rumors, with minimal difference between aggression and bogie rumors' risk levels.

**Fig. 2:**
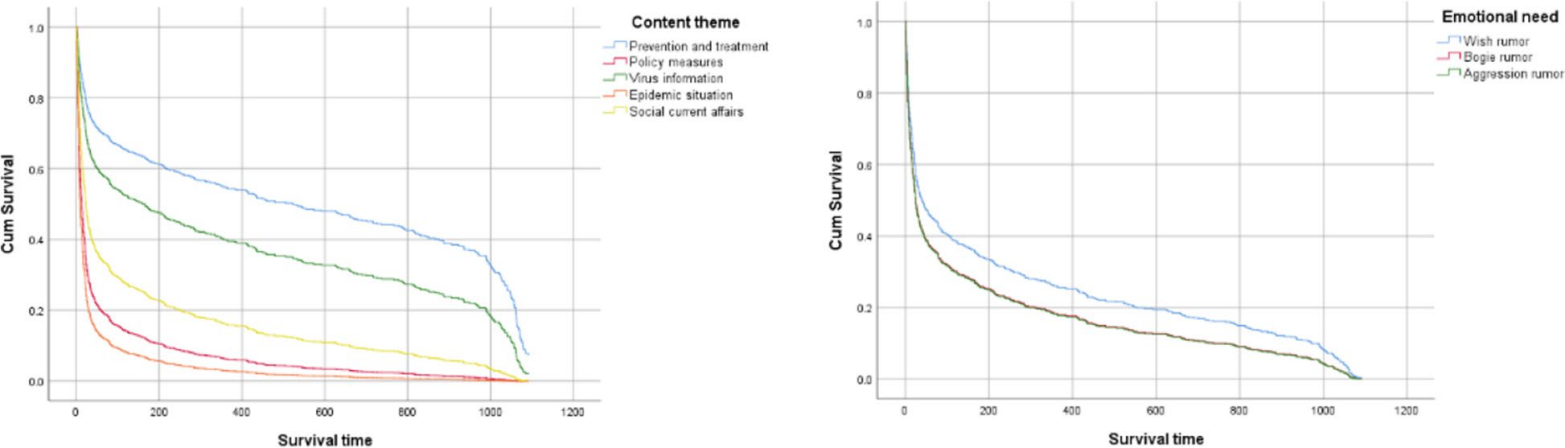
Survival curve under the Cox proportional hazards regression model

Figure 2 presents the survival curve, illustrating the time distribution of rumors while considering the influence of covariate factors. This curve offers an objective view of the time distribution relationships under the controlled conditions of other variables, utilizing the Cox proportional hazards regression model. Compared to Fig. 1, the survival function curve in Fig. 2 is smoother, reflecting the model's ability to adjust for various covariates.

Under the Cox model, there are clear disparities in the time distribution of rumors across different content themes. Rumors about the epidemic situation and policy measures are characterized by shorter survival times, whereas social current affairs rumors have a moderate survival duration. In contrast, rumors related to prevention and treatment, as well as virus information, show longer survival times. Specifically, as time (t) progresses, the survival function curves for epidemic situation and policy measures rumors show a significant downward trend. Within the first 100 days, the survival probabilities for these rumor types drop to 0.2. In contrast, the survival probability for prevention and treatment rumors remains around 0.4 even after 1000 days, followed by a sharp decline.

When analyzing the survival curves based on emotional needs, there is a notable distinction between the curve for wish rumors and those for bogie and aggression rumors. The latter two types show similar trends. According to Table 6 and controlling for the content theme, the relative hazards for bogie and aggression rumors are 0.26 and 0.27 times higher than for wish rumors, respectively. These risks are comparable, as reflected in the survival curves of Fig. 2. Among the three emotional need categories, wish rumors have the highest survival probability, with the curves for bogie and aggression rumors consistently lower.

In summary, the differences in the survival curve distributions based on content themes are more marked than those based on emotional needs. The Cox model's survival curves provide a nuanced understanding of how different factors influence the longevity of rumors, with content themes having a more pronounced effect than emotional needs.

### The three stages of duration

**Fig. 3:**
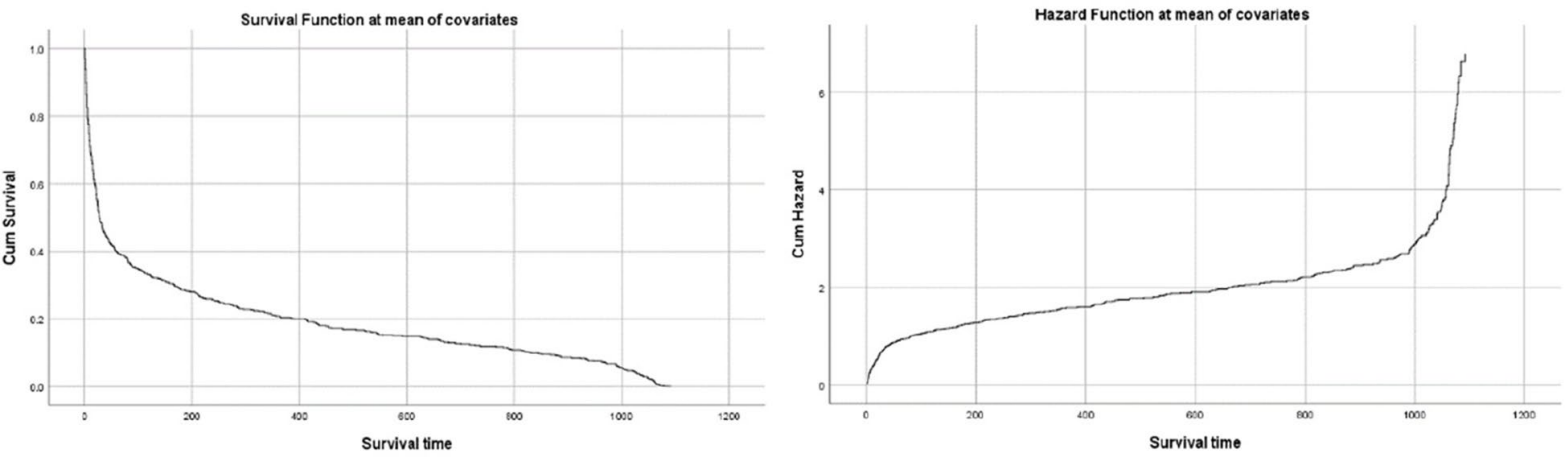
Cox model: survival and hazard at mean covariates

Figure 3 displays the survival and cumulative hazard curves at mean covariate levels, offering insights into the overall changes in the survival probability of the sample. Analyzing this figure, the study categorizes the survival time of rumors into three distinct stages: an initial rapid decline phase from 0 to 25 days, a subsequent stable phase spanning 25 to 1000 days, and a final extinction phase extending beyond 1000 days.

Figure 3 presents a detailed analysis of the survival stages of online rumors during the COVID-19 pandemic, offering an in-depth look at their complex lifecycle. The initial phase, termed the rapid decline phase (0–25 days), is marked by a sharp decrease in the survival curve, with many rumors quickly diminishing due to clear fallacies. Despite this, a subset of rumors demonstrates resilience, surviving beyond early debunking efforts. This period is characterized by a brief yet pronounced increase in the hazard curve.

The study then transitions to the stable phase (25–1000 days), where the decline in the survival curve becomes more gradual. During this phase, the probability of rumor survival decreases steadily from 50 to 10%, and the cumulative hazard curve shows a corresponding gradual increase. This phase represents a significant duration, approximately three years, indicating the prolonged presence of some rumors.

In the extinction phase (post-1000 days), there is a notable resurgence in the hazard rate, accelerating the decline of the remaining rumors. This phase is characterized by its brevity and the rapid succession in which the last few rumors disappear.

This segmentation into distinct stages challenges the conventional notion of the “Seven-day Law of Propagation” for online public opinion. The study reveals a more varied rumor lifespan, with some dissipating quickly, while others endure for extended periods. Particularly, rumors related to prevention and treatment, and virus information, show longer survival, likely due to their alignment with public interest and concerns. These insights are crucial for developing effective rumor management strategies, emphasizing the need for approaches tailored to specific content themes and emotional needs.

### The influence of different factors

This study investigates the impact of content themes on the longevity of rumors, employing Kaplan‒Meier univariate analysis and Cox proportional hazards regression models. These analyses reveal significant disparities in survival times across different content themes, indicating a need for further examination of these variations.

Rumors related to prevention and treatment typically arise from traditional remedies and historical experiences with diseases. Confronted with a novel virus and scarce information, the public often gravitates towards familiar practices, such as consuming boiled soybean water to alleviate fever. These rumors gain traction due to a cautious mindset where people prefer to be safe than sorry. In contrast, rumors involving virus information are notably harder to counter. They frequently masquerade as scientific findings, creating a semblance of credibility that can easily mislead the general populace. The selective use of research findings and the construction of causal narratives set high barriers for their debunking. In the absence of specialized platforms or expert input, dispelling these rumors poses a significant challenge. On the other hand, rumors about epidemic situations and policy measures tend to diminish more quickly. This is largely due to effective rumor management by governmental bodies. Local communities, governments, and the media place substantial emphasis on combating rumors related to epidemic updates and policy decisions. The dissemination of authoritative information through regular briefings and press conferences facilitates the swift refutation of such rumors, leading to their shorter lifespan. This efficient management highlights the critical role of government intervention in controlling rumor spread during public health crises.

Policy measure rumors often persist longer than those about epidemic situations, primarily due to the time needed for policy development and the uncertainties in announcing policies amid an evolving epidemic. These rumors cover a wide range, from regional lockdowns to major measures like constructing temporary hospitals or resuming work and school activities. Government agencies strategically evaluate whether and how to address these rumors, considering the need for detailed refutation. This careful approach enables timely policy adjustments in response to rapid changes in the epidemic, influencing the duration of these rumors.

The study reveals notable differences in the survival times of rumors associated with emotional needs, as evidenced by the Kaplan‒Meier survival estimate and the Cox proportional hazards regression model. Wish rumors, appealing to the public's desire for positive outcomes, consistently exhibit longer survival compared to bogie and aggression rumors.

Wish rumors resonate with the public's yearning for positive outcomes, particularly during challenging times. Amidst the adversities of the epidemic, these rumors provide a semblance of hope, offering psychological solace despite potential inaccuracies in the information. Such rumors persist as they reflect the enduring optimism of individuals navigating through the uncertainty and anxiety brought by the epidemic.

The dynamics of bogie and aggression rumors are similarly notable, with both types holding the potential to trigger widespread reactions that could destabilize societal harmony. Bogie rumors typically exacerbate anxiety, feeding into the existing tensions among the populace. To counter these, experts and scholars actively engage in disseminating accurate information, thus debunking sensationalized claims and alleviating public fear. For instance, rumors about drastic measures like city-wide lockdowns can disrupt daily life significantly, as seen in claims about Beijing suspending services temporarily.

In contrast, aggression rumors often arise from perceived inadequacies or mismanagement in regional epidemic responses. These rumors can fuel discontent and mistrust, potentially leading to conflicts between the public and authorities. Addressing these rumors promptly is crucial to maintaining social cohesion and ensuring the effectiveness of epidemic control measures. Both types of rumors, especially when involving specific entities or individuals, require immediate action to prevent undermining public trust and the broader efforts in managing the epidemic. The government's proactive stance in debunking such rumors is essential in mitigating their impact and preserving public order and health.

Intriguingly, the study uncovers that the credibility of the source does not markedly affect the lifespan of rumors when viewed over time. Both Kaplan‒Meier estimates and Cox model analyses underscore the minimal impact of source credibility on rumor persistence. This observation can be linked to the fluid nature of rumor sources, which may evolve over time, transitioning from highly credible origins to less reliable ones. Consequently, the public's attention often shifts more towards the content of the rumor rather than its source, treating the latter as a secondary consideration. Even among rumors originating from ostensibly credible sources, there is a notable variation in survival times, contingent on the content.

The study also examines the role of presentation format, with Kaplan‒Meier univariate analysis revealing significant differences in survival times based on the format. Image and video-based rumors tend to have similar impacts, while text-based rumors generally demonstrate extended longevity. This can be attributed to the immersive and seemingly authentic nature of visual content, which, however, provides more verifiable details. Despite this, the Cox model indicates that the overall impact of presentation format is relatively minor when all factors are considered, highlighting the predominant influence of content theme and emotional needs on rumor survival.

## Discussion

### Theoretical implications

This study undertakes a comprehensive examination of COVID-19 rumors from the perspective of survival time, delving into the distribution characteristics and determinants of rumor longevity. The primary theoretical contributions of this research are manifest in four key areas:

Firstly, this study pioneers the approach of considering rumor survival time as the dependent variable. This is a notable deviation from prior mainstream research which predominantly focused on the number or speed of rumor propagation [17, 19] or the sharing behavior of rumors [18, 20]. By concentrating on the factors influencing rumor survival time, this research offers new insights into the dissemination and persistence patterns of rumors of different types and natures, thereby enriching the academic discourse on rumor studies.

Secondly, this research innovatively identifies that rumors exhibit distinct stage characteristics based on their survival time, challenging the conventional “Seven-day Law of Propagation” [58]. We categorize the survival time of rumors into three stages: a rapid decline phase (0 to 25 days), a stable phase (25 to 1000 days), and an extinction phase (beyond 1000 days). This classification underscores the significant variability in rumor lifespans, from those that fade within 25 days to others that endure for years. The identification of these varied survival stages constitutes a potential theoretical contribution of this study.

Furthermore, the development of a comprehensive antecedents framework represents another theoretical advancement. Previous studies often addressed these factors in isolation or combined only a few elements. This study, building upon existing research, systematically examines multiple influences, including content theme, emotional appeal, source credibility, and presentation methods, and their impact on rumor survival.

Lastly, the adoption of survival analysis as a research methodology in this study is also of theoretical significance. Unlike conventional multivariate linear regression models, which frequently struggle with missing data, survival analysis adeptly handles both the outcomes and timing of survival, particularly excelling in utilizing the incomplete data inherent in censored cases. This approach allows for a more precise and unbiased representation of the survival time distribution among the sampled rumors.

### Practical implications

Several studies have analyzed that rumors detailing terrifying virus characteristics easily prompt the public to engage in irrational behaviors under heightened stress levels [59]. For example, Hu et al. [60] found that once the public perceives public health emergencies as serious, people are more likely to exhibit negative attitudes and irrational consumption behaviors, such as rushing to purchase and hoarding daily necessities. Guo et al. [61] also found that irrational behavior occurs especially in the face of sudden and destructive situations, such as the rush for Shuang Huang Lian (a traditional Chinese medicine) after the outbreak of COVID-19. The government's approach to handling rumors plays a crucial role in shaping public perception and response to these rumors. The practical applications of this study are evident in three key areas of rumor management recommendations: phased management, sustained vigilance, and typological responses.

Firstly, regarding phased management, it is imperative for the government to implement real-time monitoring and prompt debunking of rumors, tailored to their varying propagation stages. The study identifies three distinct phases in a rumor's lifecycle: rapid decline, stability, and extinction. Particularly during the initial phase, when rumors are rampant, it is essential for government agencies to leverage internet monitoring systems for early detection, engage public health authorities to issue clarifying statements, and utilize mainstream media to disseminate accurate information.

Secondly, the need for long-term attention is paramount, especially for rumors that persist over extended periods and may be overlooked yet possess significant influence. Research indicates that while some rumors may dissipate within 25 days, others can endure for years. These long-lasting rumors warrant continuous monitoring and intervention, not only at their inception but also over an extended duration.

Finally, the study advocates for typified responses, suggesting that the government and related bodies adopt varied strategies based on the nature of the rumors, encompassing their content themes, emotional triggers, and presentation styles. Findings reveal that rumors vary in longevity based on these factors. For instance, rumors with shorter lifespans require immediate action and expert involvement for refutation. In contrast, long-lasting rumors demand ongoing tracking and real-time detection. Specifically, (1) Content-wise, persistent rumors often relate to prevention, treatment, and virus information. (2) Emotionally, wish rumors tend to outlast those fueled by fear or hostility. (3) In terms of format, text-based rumors generally survive longer than those conveyed through images or videos. Consequently, relevant agencies should formulate targeted strategies to effectively counter rumors, considering their specific content, emotional appeal, and mode of presentation.

### Research limitations

While the current study offers valuable insights, it is important to recognize its inherent limitations and suggest directions for future research. One of the primary challenges relates to the data collection period, which may have led to incomplete information capture. The study's reliance on a limited set of fact-checking platforms also raises concerns about the comprehensiveness and accuracy of the data gathered. It is noteworthy that the pandemic-related rumors have been in circulation since December 2019, a timeframe not entirely encompassed in our data collection, thus potentially omitting key early-stage information. Additionally, the exclusive use of data from Tencent's “Jiao Zhen” Fact-Checking Platform poses a limitation in terms of the diversity of data sources.

Furthermore, while this research has investigated several significant factors influencing rumor dissemination, it is crucial to acknowledge that this exploration might not be exhaustive. Future studies should aim to address these gaps by extending the data collection period, diversifying data sources, and expanding the range of influencing factors examined to provide a more comprehensive understanding of rumor dynamics in the context of global health crises.

### Future research directions

The trajectory for future research can be further refined in two primary areas: data enhancement and theoretical framework expansion. Firstly, to bolster the reliability of findings, future studies should aim to extend the data collection period and incorporate a broader spectrum of data sources from various rumor detection platforms. This approach would enrich the dataset and provide a more robust basis for analysis.

Secondly, future research should embrace a diverse array of theoretical perspectives to explore additional factors that may influence the longevity and spread of rumors. This could include conducting more nuanced analyses across different media channels and demographic groups [62]. Additionally, it would be beneficial to consider the individual characteristics of the public along with other socio-cultural elements [63]. An exploration within the “4I” framework, encompassing the information itself, individual characteristics, interpersonal or community dynamics, and institutional factors [64], could also provide insightful perspectives. By broadening the scope of data sources and theoretical approaches, future research can significantly advance our understanding of rumor dynamics, particularly in the context of rapidly evolving public health scenarios.

## Conclusion

This investigation explores the complex mechanisms underlying the lifespan of rumors, identifying a three-phase trajectory: initial rapid decline, subsequent stabilization, and eventual extinction. Utilizing the Kaplan‒Meier estimator, it intricately examines how content themes, emotional needs, and presentation formats intertwine to shape rumor survival. Notably, while source credibility influences perception, it does not significantly impact rumor longevity. The study categorizes various rumor themes based on their survival probability, with prevention and treatment, virus information, and social current affairs rumors displaying more resilience compared to policy measures and epidemic situation rumors.

The Cox proportional hazards regression model further elucidates the roles of content themes and emotional needs in influencing rumor survival. The analysis reveals significant variations in survival times across different themes, with epidemic situation, policy measures, and social current affairs rumors showing a markedly higher propensity for early dissipation. Similarly, emotional needs influence the persistence of rumors, with wishful content demonstrating more longevity compared to bogie and aggression-themed rumors.

This research underscores the lesser impact of presentation format compared to content themes and emotional needs. It highlights the complexities involved in rumor propagation and survival, necessitating multifaceted strategies for effective rumor management. The study advocates for targeted approaches by governmental agencies to address rumors, emphasizing the need for real-time debunking and societal collaboration in rumor refutation. It also calls for the enhancement of information dissemination channels and communication mechanisms to counter rumor genesis. Experts and scholars are encouraged to contribute by disseminating accurate health information, and media platforms are urged to play a crucial role in scrutinizing information veracity and enhancing public media literacy. This comprehensive approach is pivotal in managing rumors effectively, particularly in the context of public health crises.

## Data Availability

The selection of rumor events comes from the Tencent “Jiao Zhen” Verification Platform’s “Real-Time Debunking of Novel Coronavirus Pneumonia” system (website link: https://vp.fact.qq.com/home), publicly available. Based on these events, we then used Python to crawl data of variables on the internet. The data analysed during the current study available from the corresponding author on reasonable request.
